# Health promotion through structured oral hygiene and good tooth alignment

**DOI:** 10.3205/dgkh000411

**Published:** 2022-05-10

**Authors:** Axel Kramer, Christian Splieth

**Affiliations:** 1 Institute of Hygiene and Environmental Medicine, University Medicine Greifswald, Greifswald,Germany; 2 Preventive & Pediatric Dentistry, University Medicine Greifswald, Greifswald, Germany

**Keywords:** periodontitis, gingivitis, caries, systemic effects, prevention, oral hygiene, aligner

## Abstract

**Aim:**

Periodontal diseases and caries are two of the most common forms of chronic degenerative diseases, with consequences not only for the oral cavity manifesting as tooth loss, orofacial pain and xerostomia, but also with effects on the cardiovascular system and, in the elderly, on the pneumonia rate. This can be prevented or controlled by structured oral hygiene.

**Method:**

Based on a systematic literature search in PubMed, the possibilities for ensuring structured oral hygiene are analyzed.

**Results and conclusion:**

Limiting the consumption of sugary meals and beverages, regular removal of food debris – supplemented by sugar-free chewing gum if desired – and preventing plaque formation by brushing with fluoridated toothpastes, using dental floss and interdental brushes after meals, serve to prevent or control gingivitis, periodontitis and caries. In the long term, the development of periodontitis-associated cardiovascular diseases and, in the elderly, the risk of pneumonia can probably be reduced. Antiseptic rinsing of the oral cavity is an important supplement to prevent periodontitis, especially in cases of limited ability to perform mechanical biofilm removal, but also for the prevention of respiratory infections. Proper functional tooth alignment is important for optimal mechanical cleaning to prevent plaque accumulation. If correction of misaligned teeth is possible with the use of removable aligners instead of fixed orthodontic appliances, these are to be preferred because of the better accessibility for mechanical hygiene measures.

## Zusammenfassung

**Zielsetzung:**

Parodontopathien und Karies zählen zu den häufigsten Formen chronisch degenerativer Krankheiten mit Auswirkungen nicht nur in der Mundhöhle mit Zahnverlust, orofazialen Schmerzen und Xerostomie, sondern auch auf das kardiovaskuläre System und im Senium auf die Pneumonierate. Dem muss durch strukturierte Mundhygiene entgegengewirkt werden.

**Methode:**

Auf der Grundlage einer systematischen Literaturrecherche in PubMed sollten die Möglichkeiten zur Gewährleistung einer strukturierten Mundhygiene analysiert werden.

**Ergebnisse und Schlussfolgerungen:**

Die Einschränkung des Konsums zuckerhaltiger Mahlzeiten und Getränke, die regelmäßige Entfernung von Speiseresten, ggf. ergänzt durch Kaugummikauen, und die Verhinderung der Plaquebildung durch Zähneputzen mit fluoridhaltigen Zahnpasten, Anwendung von Zahnseide und Interdentalbürsten nach den Mahlzeiten dienen der Verhinderung von Gingivitis, Parodontopathien und Karies. Langfristig kann dadurch vermutlich auch mit Periodontitis assoziierten systemischen Erkrankungen vorgebeugt werden. Insbesondere bei eingeschränkter Fähigkeit zur Durchführung der mechanischen Biofilmentfernung, aber auch zur Prävention respiratorischer Infektionen, ist die antiseptische Spülung der Mundhöhle eine wichtige Ergänzung zur Prävention der Periodontitis. Eine gute, funktionale Zahnstellung erleichtert die mechanische Reinigung zur Verhinderung der Plaqueakkumulation. Sofern mit dem Einsatz von Alignern anstelle festsitzender kieferorthopädischer Apparaturen die Stellungskorrektur möglich ist, sind diese wegen der besseren Zugängigkeit für mechanische Hygienemaßnahmen zu bevorzugen.

## Introduction

Periodontal diseases and caries are among the most common forms of inflammatory and infectious diseases and one of the most common forms of chronic degenerative disease [1], [2]. With increasing life expectancy, the number of people with chronic diseases, immune deficiencies and impairments leading to multimorbidity and need for long-term care is increasing. With increasing age, the risk of diseases of the oral cavity involving the periodontium and the teeth also increases. Different periodontal diseases resulting in tooth loss affect the well-being, psychological condition, social relationships and physical health. The quality of life is positively associated with a higher number of teeth and the chewing function they provide, greater number of occlusal pairs, implant-supported overdentures, and the short dental arch concept, but negatively associated with xerostomia, orofacial pain, and poor chewing ability [3]. However, subjectively perceived esthetics in the oral and facial region is also important for the quality of life and psychosocial well-being [4]. Due to growing evidence on the importance of the oral cavity for health and well-being, the maintenance and promotion of oral-cavity health are gaining importance beyond the improvement of the local situation in the oral cavity. In terms of health policy, it is crucial to start preventing gingivitis, periodontitis, caries, and ensuring good tooth position by beginning oral care in early childhood rather than at the onset of problems. 

Considering the current state of knowledge on the interrelationship between the health of the oral cavity and the effects on the rest of the organism, the following highlights ways to maintain oral-cavity health.

## Methods

Based on a systematic literature search in PubMed, the possibilities of ensuring structured oral hygiene are analyzed with following search criteria: Periodontitis, gingivitis, caries, combined with prevention, oral hygiene, periodontitis-associated cardiovascular diseases, periodontitis-associated pneumonia, systemic effects of oral health, senescence and oral health, influencing factors on oral health, fixed orthodontic appliances, wafer-thin, aligner, combined with oral health, antiseptic mouth rinse oral health. 

## Results

### Influence of pregnancy and senescence on oral cavity health

Pregnancy is associated with an increased caries and periodontal risk. By constructing a cariogram, it is possible to identify risk factors for the development of caries as a basis for intensified prevention [5]. Thus, intensified oral hygiene measures are advisable.

With increasing age, physiological, hormonal and cellular changes occur. In principle, all cells can be affected by aging processes. If, for example, the number of T lymphocytes decreases, this results in an increased susceptibility to infection. Furthermore, the capacity of the immune system is reduced by unfavorable lifestyle factors, such as smoking, alcohol consumption, inactivity, and unhealthy diet, whereas regular physical activity is associated with an improvement in the immune response even at older ages [6]. An important feature of senescence is the transformation of somatic cells into a “senescence-associated phenotype” associated with the production of inflammatory mediators [7]. In the context of aging, the chronic burden of inflammatory mediators (interleukins, acute phase proteins, etc.) is called “inflammaging” [8].

### Association of oral cavity health with systemic diseases

Observational studies support the hypothesis of a weak association between periodontitis and cardiovascular disease (CVD). However, whether poor periodontal health causes CVD is still unclear. Masi et al. [9] concluded that cardiovascular prevention begins in the oral cavity. Furthermore, periodontal microbes are associated with atherosclerosis, hypertension, and dyslipidemia, but again, causality is still unproven [10].

Epidemiologic studies suggest an association between oral health and pneumonia in older people in outpatient or inpatient medical care. Of seniors living independently at home, 13% were hospitalized for pneumonia within 10 years. Approximately 22% of the disease burden can be explained by high plaque levels and limited physical mobility, independent of other confounding variables [11]. These data underscore the importance of oral hygiene [12].

There is also an association between periodontitis and metabolic as well as psychiatric diseases, probably based on inflammatory processes [13].

### Prevention of periodontitis and caries

#### Oral hygiene

In addition to subjective well-being, the objective of oral hygiene is to maintain an intact mucous membrane, a plaque-free tongue, the dental status and supple lips. Inadequate care can lead to dehydration, rhagades, aphthae, damage to the teeth, mucositis, gingivitis, periodontitis, parotitis, and possibly also to descending respiratory infections and cardiovascular side effects. Especially at risk are disabled patients, seniors, patients undergoing chemotherapy or radiotherapy, and immunosuppressed patients.

Caries-protective nutrition [14] and consistent, structured oral hygiene [15] will counteract the risk of microbially maintained inflammation in the oral cavity, including systemic side effects emanating from it [16]. Oral hygiene includes regular removal of food debris and mechanical removal of biofilm for plaque prevention, with the aim of preventing gingivitis, periodontitis and caries [14], [15]. In the long term, this is likely to prevent systemic disease, as periodontal inflammation exacerbates systemic inflammation [10], [13]. Intensified oral hygiene (toothbrushing, supragingival scaling) has been shown to permanently improve oral inflammatory status and slow periodontal deterioration in patients with type 2 diabetes [17]. If patients are unable to perform oral hygiene adequately, care takers should be instructed and trained to guarantee standard oral hygiene procedures.

For prophylaxis of caries, gingivitis and periodontitis, with maintenance of the physiological oral cavity flora, at least twice-daily toothbrushing with a fluoridated toothpaste using an electric toothbrush, if possible [18], is effective, provided the gums are intact. Brushing only once a day has a lower caries-preventive effect [19], [20]. There is no epidemiological evidence on the optimal brushing duration. The only evidence is that 1-minute toothbrushing removes an average of 27% of plaque, and 2-minute toothbrushing removes 41% of plaque [21]. A longitudinal cohort study demonstrated an association between the frequency of toothbrushing and the development of new carious lesions in children aged 6–10 years [22]. Toothbrushing should be renewed after 8 to12 weeks. The use of dental floss and interdental brushes of different thicknesses for the interdental spaces additionally prevents the accumulation of biofilm. Brushes angled in the bristle area facilitate cleaning of the posterior teeth. Dentures, fixed orthodontic appliances and aligners should be included in oral hygiene. It is important to practice an effective brushing technique with a systematic approach [14]. Particularly in cases of increased caries risk during pregnancy and diseases with altered oral cavity flora, tooth cleaning after every meal is especially important, if possible in conjunction with the use of dental floss. Unfortunately, brushing teeth in the morning and evening is not universal practice. Brushing teeth is especially important before going to bed, because the reduced saliva flow at night enhances bacterial reproduction and attacks on the tooth enamel. 

In additional to fluoridated toothpaste, fluoridated table salt should be used [14]. In cases of increased caries risk, e.g., during pregnancy, it is advisable to use toothpastes with increased fluoride concentration or fluoride varnishes, gels or antiseptic mouthrinses to remineralize the enamel [14], [23].

If tooth cleaning is supplemented by antiseptic mouth rinses, which is particularly useful if the ability to perform mechanical biofilm removal is limited, active ingredients with the risk of developing resistance, including the risk of cross-resistance to antibiotics, should not be used. This applies to the antiseptic agents chlorhexidine digluconate [24], [25] and triclosan [26], [27], commonly used in mouthrinses, whereas essential oils are not known to develop resistance [28], [29]. Regular antiseptic oral cavity rinsing (2–3 times daily) additionally reduces the frequency and severity of infections during epidemic or pandemic periods of respiratory infections, demonstrated for influenza and COVID-19 [30], [31].

The number of sugary meals and snacks, including sugary drinks, should be kept to a minimum. Food and beverages without free sugars are to be preferred, especially in between meals. Regular chewing of sugar-free chewing gum can help prevent caries and is therefore particularly useful after meals [14].

### Ensuring good tooth alignment

Good tooth alignment improves function and self-cleaning and support effective oral hygiene, because mechanical tooth cleaning can only be effective where it reaches the tooth. Preventing biofilm formation in the interdental spaces is crucial for the long-term prevention of periodontitis. There are indications that the orthodontic treatment of malocclusions possibly could coincide with a reduced risk of compromised oral health [32] and periodontitis prevalence [33]. To restore good, functional tooth position, fixed orthodontic appliances or wafer-thin, transparent removable dental splints that rest on the teeth (so-called aligners) can be used. The disadvantage of fixed appliances is the difficulty in ensuring oral hygiene due to numerous surfaces that are difficult to reach [34]. In contrast, aligners facilitate oral hygiene [35] and have been shown to significantly improve periodontal health indices, although the strength of evidence from studies is moderate [36], [37], [38], [39]. However, the indication for aligners must be carefully considered, as not all tooth movements are possible with aligner therapy. For buccolingual inclination of the maxillary and mandibular incisors with mild to moderate tooth misalignment, results comparable to those of fixed appliances can be achieved [37]. When comparing results with aligners vs fixed braces, both were effective, with aligners having the advantage of segmented movement of the teeth and, depending on the malocclusion, shortened treatment time; however, aligners were not as effective as braces in establishing adequate occlusal contacts and controlling the torque of the teeth [40]. In conclusion, aligners are not a full substitute for orthodontic treatment in childhood and adolescence, but when used as indicated, they improve the guarantee of good dental hygiene and thus contribute to tooth preservation. Their use should therefore be reviewed, particularly in cases of incipient periodontitis.

## Conclusions

The aim of oral hygiene is tooth preservation by dental health maintenance or its restoration in case of caries, using home and individual professional dental prophylaxis. In the long term, the development of periodontitis-associated cardiovascular diseases and, in the elderly, the risk of pneumonia can probably be reduced.

## Notes

### Competing interests

The authors declare that they have no competing interests.

### Funding

The authors received no funding.
